# Complete Genome Sequence of the Bacterium Aalborg_AAW-1, Representing a Novel Family within the Candidate Phylum SR1

**DOI:** 10.1128/genomeA.00624-15

**Published:** 2015-06-11

**Authors:** Morten Simonsen Dueholm, Mads Albertsen, Mikkel Stokholm-Bjerregaard, Simon J. McIlroy, Søren M. Karst, Per Halkjær Nielsen

**Affiliations:** Center for Microbial Communities, Department of Chemistry and Bioscience, Aalborg University, Aalborg, Denmark

## Abstract

Here, we present the complete genome sequence of the candidate phylum SR1 bacterium Aalborg_AAW-1. Its 16S rRNA gene is only 85.5% similar to that of the closest relative, RAAC1_SR1, and the genome of Aalborg_AAW-1 consequently represents the first of a novel family within the candidate phylum SR1.

## GENOME ANNOUNCEMENT

Community analyses of environmental microbial communities using 16S rRNA sequencing have revealed a broad diversity of previously unknown microorganisms. Many of these microorganisms belong to phyla without any cultivated representatives (1). These are known as “candidate” phyla (2). Obtaining complete genome sequences of microorganisms within these candidate phyla will extend our understanding of bacterial evolution and provide some of the first insights into their physiology and ecology.

Members of the candidate phyla SR1 are frequently reported in microbial community studies based on 16S rRNA sequencing (3). Presently, there exists only one complete (4) and two partial genomes (5, 6) from the candidate phylum SR1. These genomes are relatively small (approximately 1 Mbp), with their preliminary annotation suggesting a nonrespiring anaerobic fermentative metabolism and possible involvement in the hydrolysis of complex organic material (4–6). The sequencing of more SR1 genomes will reveal more features of the physiology of this candidate phylum.

The candidate phylum SR1 bacterium Aalborg_AAW-1 was identified within a laboratory-scale enrichment reactor that was seeded with activated sludge from Aalborg West wastewater treatment plant (Aalborg, Denmark) and operated to simulate the enhanced biological phosphorus removal (EBPR) process.

Metagenomic DNA was isolated from the biomass of the laboratory-scale enrichment reactor using the FastDNA SPIN kit for soil (MP Biomedicals). Paired-end sample libraries were prepared using both Nextera and TruSeq PCR-free kits (Illumina, Inc.). A mate pair library was generated using the Nextera mate pair kit (Illumina, Inc.) with the gel-free approach. The prepared libraries were sequenced using either an Illumina MiSeq or a HiSeq 2000 sequencer (Illumina, Inc.). Paired-end and mate pair reads were trimmed and quality checked using NextClip (7) and CLC Genomics Workbench version 7.0 (CLC bio-Qiagen), as previously described (8). All trimmed metagenome reads were assembled using the CLC *de novo* assembly algorithm (*k*-mer, 63; scaffold length, ≥1 kbp). The SR1 genome was extracted using the mmgenome R package (http://madsalbertsen.github.io/mmgenome/) that relies on the differential coverage principle (9). Gaps were closed and subsequently validated by manual read mapping in CLC Genomics Workbench. The average coverage of the assembly was 40×. Genome annotation was carried out using Prokka (10) using the genetic code 25 required for candidate SR1 bacteria (6).

The complete genome is composed of a circular chromosome of 1,044,756 bp. The overall G+C content is 33.3%. The strain is most closely related to candidate phylum SR1 bacterium RAAC1_SR1 (4), with which it shares 85.5% 16S rRNA sequence identity. Therefore, Aalborg_AAW-1 is suggested to be the first sequenced representative of a novel family within the candidate phylum SR1 (11). The average nucleotide identity in BLAST (ANIb) between Aalborg_AAW-1 and RAAC1_SR1 was only 65.9% (4, 12). Prokka annotation identified 994 coding sequences (CDSs), three rRNA (16S, 5S, and 23S) genes, and 38 tRNA genes. The annotation supports a nonrespiring anaerobic fermentative metabolism coupled with the hydrolysis of complex organic material, in line with other SR1 bacteria. However, in contrast to other SR1 bacteria, Aalborg_AAW-1 does not encode the archaeon-type ribulose-1,5-bisphosphate carboxylase (RuBisCO) proposed to be involved in AMP recycling (4–6).

### Nucleotide sequence accession number.

The whole-genome sequencing project has been deposited at GenBank under the accession no. CP011268.
